# The influence of probiotic bacteria and human gut microorganisms causing opportunistic infections on *Blastocystis* ST3

**DOI:** 10.1186/s13099-019-0287-8

**Published:** 2019-02-14

**Authors:** M. Lepczyńska, E. Dzika

**Affiliations:** Department of Medical Biology, Faculty of Health Sciences, Collegium Medicum, University of Warmia and Mazury, Żołnierska 14C, 10-561 Olsztyn, Poland

**Keywords:** *Blastocystis*, Subtype 3, Eradication, Probiotics, Gut microbiota

## Abstract

**Background:**

*Blastocystis* subtype 3 is an intestinal protist present in humans throughout the world with a controversial pathogenic potential. It has been suggested that probiotic bacteria inhibit the multiplication of gut protozoans, while others are beneficial for their development. This study aimed to evaluate the efficacy of the lactic acid bacteria *Lactobacillus rhamnosus*, *Lactococcus lactis* and *Enterococcus faecium* in *Blastocystis* ST3 eradication and the relevance of the intestinal microorganisms *Escherichia coli*, *Candida albicans* and *Candida glabrata* in protozoan proliferation. *Blastocystis* xenic and axenic culture was co-incubated with the above-mentioned microorganisms and their cell free supernatants at different concentrations in vitro. The number of protozoan cells was counted every day.

**Results:**

Both experiments, with xenic and axenic cultures, showed *Blastocystis* inhibition by *L. rhamnosus* and *L. lactis* and their supernatants from the 2nd day of co-incubation. Furthermore, co-incubation with both *E. faecium* and *E. coli* showed a beneficial influence on *Blastocystis* during the first 2 days. Only after 3 days did the above-mentioned bacteria start to inhibit *Blastocystis* growth in both cultures. The supernatant containing the metabolites of *E. coli* was effective to a lesser degree. Compared to the control samples, co-incubation with both *C. albicans* and *C. glabrata* showed a faster decrease in *Blastocystis* proliferation, but this was not statistically significant.

**Conclusions:**

This study has shown the potential of using *L. rhamnosus* and *L. lactis*, as well as *E. faecium* as a prophylactic treatment against *Blastocystis* colonization or as an additional treatment regimen in combination with standard drugs.

## Background

*Blastocystis* is a protist present throughout the world in the intestines of both healthy and symptomatic humans and animals [1, 2]. Its pathogenic potential is still controversial. This unicellular microorganism causes gastrointestinal as well as skin disorders [3, 4]. Seventeen morphologically indistinguishable subtypes have been identified based on an analysis of a small subunit rDNA (SSU rRNA) gene sequence among *Blastocystis* isolated from humans and animals. It has been suggested that ST3 may be the only subtype (ST) of human origin [5]. That is why this subtype was chosen for analysis in this study. The fecal–oral route is most likely the main mode of transmission. Children, the elderly and immunocompromised individuals appear to be highly susceptible to *Blastocystis* invasion [6], while other researchers have suggested that people between 30 and 50 years of age are most prone to being infected by *Blastocystis* [7–10]. In the recent literature, researchers have been discussing the correlation between different *Blastocystis* subtypes and their pathogenic potential. The explanations for pathogenicity may include intra-subtype variations in *Blastocystis* protease activity, or differences in the intestinal microbiota of the individual host, which can interact to mediate host colonization and *Blastocystis* virulence [11, 12]. Recently, it has been found that the presence of gut microbiota seems to be essential for the pathogenic expression of enteric protozoan such as *Blastocystis*. Berrilli et al. [13] suggest the hypothesis that some intestinal bacteria inhibit multiplication of gut protozoa.

A 2014 study by Nourrisson et al. [14] and a 2016 study by Nagel et al. [15] suggest that *Blastocystis* may be used as an indicator of microbiota changes—a lower abundance of *Faecalibacterium prausnitzii* and *Bifidobacterium* spp. was reported to lead to the intestinal dysbiosis. On the other hand, in 2016, Audebert et al. [16] suggested that colonization by *Blastocystis* could be associated with healthy gut microbiota. Their study showed a higher bacterial diversity in *Blastocystis*-colonized patients compared to that identified in *Blastocystis*-free individuals. However, the same study showed an increasing level of the *Lactobacillaceae* family in patients not colonized by *Blastocystis*. Many researchers have reported on the inhibition of a wide range of pathogenic microorganisms like *Giardia* sp., *Entamoeba histolytica*, *Eimeria* sp. or *Cryptosporidium parvum* by probiotic bacteria [17–23]. Also there have been previous studies which have shown the effects of certain probiotic yeasts—*Saccharomyces boulardii* on *Blastocystis* development [24].

The most recent results of the latest studies leave the pathogenicity of *Blastocystis* still unclear. Researchers still do not know if *Blastocystis* is an agent of gut dysbiosis and is responsible for changing the microbiotic diversity, or if the metabolic dysfunctions and changes in the content of microbiota are the reason for the higher colonization by *Blastocystis*. There is a possibility that some species of bacteria are creating the conditions for *Blastocystis* colonization in the gut. It may also depend on the parasitic subtype [16].

The World Health Organization (WHO) defines probiotics as “live organisms which when administered in adequate amounts confer a health benefit to the host” [25]. As an alternative bio-therapeutic for giardiosis, amoebiasis or cryptosporidiosis, there are a number of studies which have been conducted. In our study, we have aimed to explore the inhibitory effect of 3 different probiotics and 3 species causing opportunistic infections on *Blastocystis* proliferation for the first time.

## Materials and methods

### *Blastocystis* cultures

*Blastocystis* subtype 3 was kindly provided by C. Rune Stensvold (Statens Serum Institute, Copenhagen, Denmark) and cultured in modified Jones’ medium (pH = 7.1) [mix of 93.8 mL Na_2_HPO_4_—9.46 g/L of distilled water, 31.3 mL KH_2_HPO_4_—9.08 g/L of distilled water, 562.5 NaCl—9 g/L of distilled water, 0.1% of yeast extract (Oxoid, UK)] supplemented with 10% horse serum (Sigma-Aldrich, USA) [26, 27] at 37 °C in tightly closed polypropylene 12 mL Falcon tubes, in anaerobic conditions. Because the experiment was performed in two ways, two versions of *Blastocystis* culture—xenic and axenic were conducted. The xenic culture (containing bacteria from the patient gut) was subcultured every 2–3 days. The axenic culture (without bacteria) was obtained by supplementation with 100 UI/mL penicillin and 100 μg/mL streptomycin (Sigma-Aldrich, USA) and incubated for 3–4 days. The cultures were then screened using standard microscopy [28].

### Bacterial and fungal isolates and growth conditions

A lyophilized stock of the organisms was obtained from the American Type Culture Collection (ATCC). The commensal bacteria *Lactobacillus rhamnosus* (ATCC 7469), *Lactococcus lactis* (ATCC 11454), *Enterococcus faecium* (ATCC 6057), and the microorganisms causing opportunistic infections *Escherichia coli* (ATCC 25922) as well as *Candida albicans* (ATCC 64548) and *Candida glabrata* (ATCC 15126) isolates were used in the present study. All isolates were previously purchased in MicroSwab form from Merck (Warsaw, Poland). The bacterial and fungal isolates were freshly cultivated on Tryptone Soy Broth (TSB) (Merck, Poland) before the experiments and also stored in TSB with 20% glycerol at − 70 °C until needed. The bacteria were routinely cultured on TSB (pH 7.3) for 2 days at 37 °C and the fungi were cultured on Sabouraud broth (pH 5.8) for 6 days at 24.5 °C. Sabouraud broth contains mycological peptone (10 g/L), glucose (20 g/L). All of the microorganisms were incubated in tightly closed polypropylene 12 mL Falcon tubes, in anaerobic conditions.

### Bacteria preparation

Each isolate of bacteria was harvested by centrifugation (5525×*g*, 15 min) from TSB after 2 days of incubation and washed three times with sterile PBS (phosphate buffered saline, pH 7.0). The pellet was suspended in sterile Jones’ medium [26, 27]. The optical density (OD^620^) of the bacterial suspensions was adjusted to 0.5 ± 0.06, 1.0 ± 0.06, and 1.5 ± 0.06 in Jones’ medium. Aliquots of the bacterial suspensions were diluted to 1:100, 1:1000, 1:10000 with PBS. 50 µL from each dilution was spread on Tryptone Soy Agar (TSA) plates. Plates were incubated at 37 °C for 2 to 4 days and colonies counted. Final concentrations of bacterial suspensions are presented in Table 1.

**Table 1:** Concentrations of microorganisms used to the experiment

Bacteria/fungi	OD^620^; colony forming units (CFU)/mL		
	Concentration I	Concentration II	Concentration III
*Escherichia coli*	0.54; 4.48 × 10^8^	1.04; 8.56 × 10^8^	1.49; 1.22 × 10^9^
*Enterococcus faecium*	0.51; 4.02 × 10^8^	1.06; 8.48 × 10^8^	1.51; 1.23 × 10^9^
*Lactobacillus rhamnosus*	0.56; 4.48 × 10^8^	1.05; 8.72 × 10^8^	1.52; 1.25 × 10^9^
*Lactococcus lactis*	0.55; 4.40 × 10^8^	1.06; 8.48 × 10^8^	1.50; 1.22 × 10^9^
*Candida albicans*	1.75 × 10^4^	2.85 × 10^5^	1.85 × 10^6^
*Candida glabrata*	1.55 × 10^4^	2.7 × 10^5^	1.8 × 10^6^

### Fungi preparation

Two isolates of fungi, *Candida albicans* and *Candida glabrata*, were harvested by centrifugation at 2300×*g* for 10 min and washed three times in sterile PBS. Subsequently, the fungi were concentrated into pellet form by centrifugation and suspended in Jones’ medium. The number of fungi cells was determined by counting in a Neubauer chamber (Heinz Herenz, Hamburg, Germany), and adjusted to the final concentrations (Table 1).

### Preparation of cell free supernatants (CFS)

Cell free supernatant is a suspension of microorganism metabolites of chemical compounds. CFS of bacteria was obtained from TSB broth cultures after 21 h incubation at 37 °C by centrifugation at 4000×*g* for 10 min. Supernatant was filtered through 0.20 µm pore size filters (SARSTEDT AG & Co. KG, Germany) [22]. The CFS of fungi was obtained from cultures on Sabouraud broth after 5 days of incubation at 24.5 °C by centrifugation at 2300×*g* for 10 min. The supernatant was sterilized by filtration with a 0.20 µm syringe filter. The pH of all supernatants was measured.

### Metronidazole preparation

A stock solution of metronidazole (MTZ, Sigma-Aldrich, USA), as a reference antiprotozoan drug [29] was prepared by adding 50 mL of sterile distilled water to 3000 mg of the drug to give a final concentration of 60 mg/mL. This was stored in a dark bottle at 4 °C [30]. MTZ was prepared at different concentrations directly before use in the experiment.

### Experimental setup and procedure

The number of *Blastocystis* cells after 2 days of laboratory incubation used for the experiment was determined by counting in a Neubauer chamber. A final concentration of *Blastocystis* in Jones’ medium was approximately 2.8 × 10^5^ cells/mL for the fungi experiment, 2.9 × 10^5^ cells/mL for the bacterial influence assay and 2.5 × 10^6^ CFU/mL for the experiment using cell free supernatants. Sterile 5 mL polypropylene tubes (Equimed, Poland) were used. One milliliter of each microorganism described above and its CFSs were inserted into 5 mL tubes (Equimed) containing 3 mL of Jones’ medium and 1 mL of *Blastocystis* xenic as well as axenic cultures in triplicate and the tubes were then sealed with a lid. Four milliliter of Jones medium with 1 mL of *Blastocystis* culture were used as control samples (pH = 6.28) in the experiment containing alive microorganisms. For the CFS assay as control samples 1 mL of *Blastocystis* culture, 3 mL of Jones’ medium and 1 mL of TSB (pH = 6.25) or Sabouraud broth (pH = 3.97) were used. The reference antiprotozoal drug metronidazole (MTZ) was tested against *Blastocystis* using three different concentrations—1 µg/mL, 5 µg/mL, and 10 µg/mL. The parasite was co-incubated for 5 days with each species of bacteria, and 7 days with each species of fungi because *Candida* sp. is a yeast which needs more time to multiply. *Blastocystis* was also co-incubated with supernatants of each microorganism for 6 days at 37 °C statically, in tightly closed polypropylene 5 mL tubes, in anaerobic conditions. Each day of incubation the number of *Blastocystis* cells was determined by counting in a Neubauer chamber. The viability of *Blastocystis* cells was assessed by using staining with 0.4% Trypan blue solution. Unstained cells were counted. The pH was measured every day of co-incubation. All experiments were repeated three times.

The influence on *Blastocystis* was determined by calculating the mean and SD (standard deviation) of the number of parasite cells in the 5 mL tubes with microorganisms and the mean of number of *Blastocystis* cells in the control samples. A cell count was taken for each bacterial and fungal concentration as well as for cell free supernatants. The results were reported as an average.

### Statistical analysis

The number of viable morphological forms of *Blastocystis* in treatment and controls were compared using *t*-test (GraphPad Prism 7.04), as well as Pearson Chi square and two-way ANOVA tests were used whenever appropriate. To compare the influence of the dilutions according to the time of co-incubation, three-way ANOVA (Tukey’s test) was used. A *p* value of < 0.05 was considered as statistically significant.

## Results

### Co-incubation of bacteria with *Blastocystis* ST3 xenic culture

**Fig. 1:**
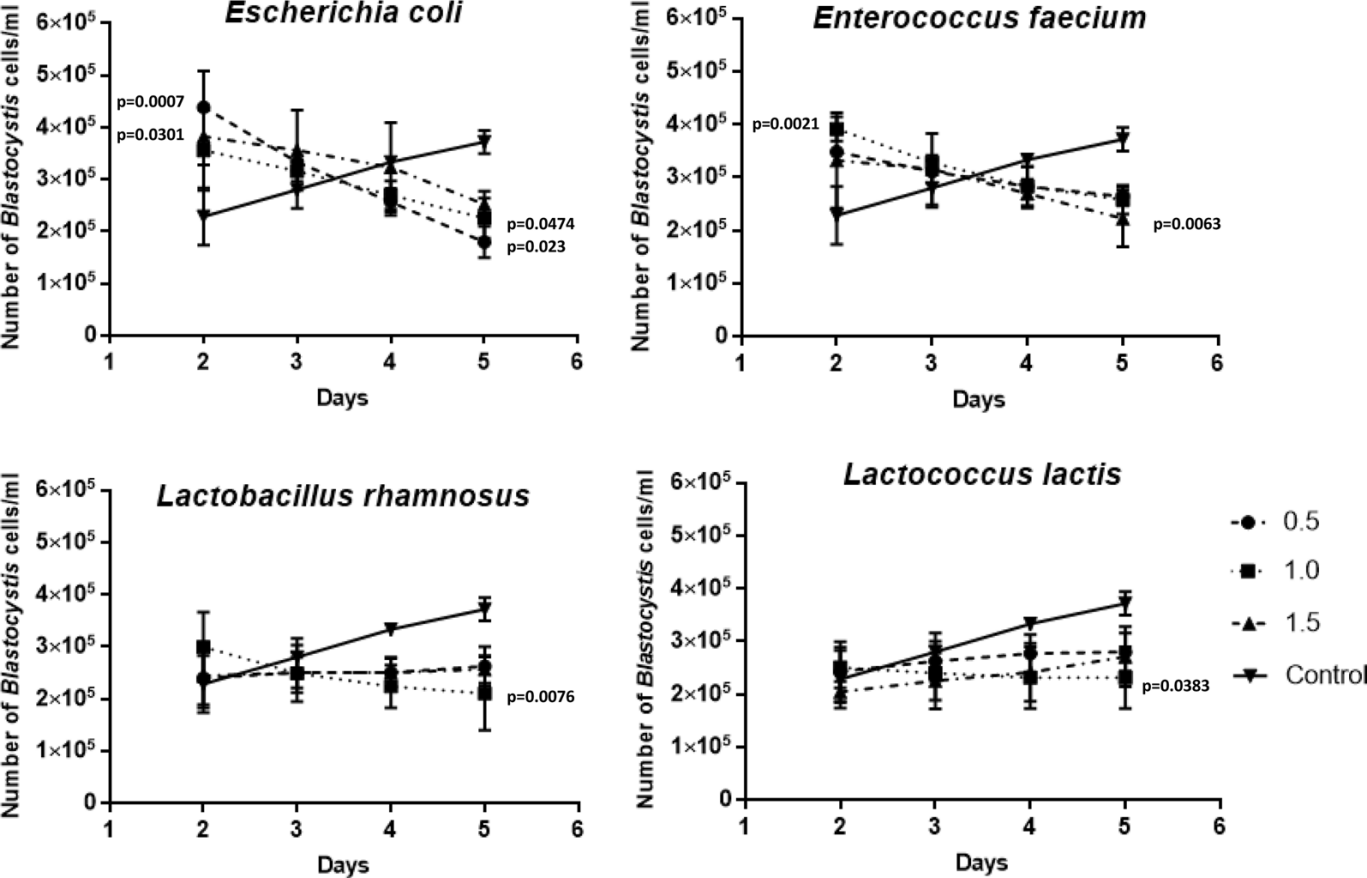
The influence of different concentrations OD^620^ = 0.5, 1.0 and 1.5 of chosen bacteria on *Blastocystis* xenic culture development according to time of co-incubation

The antiparasitic activity of the chosen bacteria against *Blastocystis* ST3 was investigated in vitro. The results of this study showed that the bacterial inoculum had an influence with regards to the protozoan, but the effectiveness of some of them is more intense than the others. The mean and standard deviation values were calculated with respect to the cell counts of the control. In control samples, *Blastocystis* proliferation was observed from 2 to 5 days. The total viable *Blastocystis* cells in bacteria-treated cultures were counted every day starting from the 2nd day of co-incubation when the number of parasites increased significantly after the addition of different concentrations of *E. coli* and *E. faecium* (Fig. 1). An inhibition of *Blastocystis* division was observed after 3 days and later of co-incubation with *L. rhamnosus* and *L. lactis* (Fig. 1). In this case, the number of *Blastocystis* cells co-incubated with *E. coli* and *E. faecium* was similar to the control samples. Furthermore, after 4 and 5 days *E. faecium* and *E. coli* had a negative influence on *Blastocystis* proliferation, and that inhibition was statistically significant.

The influence of different bacterial concentrations was statistically analyzed. The number of added *E. coli* cells when the optical density (OD^620^) was 0.5 and 1.5 (4.48 × 10^8^ CFU/mL and 1.22 × 10^9^ CFU/mL, respectively) had a significant influence on increased *Blastocystis* proliferation during the first 2 days (p = 0.0007 and p = 0.0301) as compared to the control sample. As mentioned above, the number of parasites decreased on the 5th day of co-incubation with *E. coli*. In this case, the concentration OD^620^ = 0.5 again had a significant influence (p = 0.023). The OD^620^ = 1.0 concentration also inhibited protozoan proliferation (p = 0.0474). Similar results were observed with regards to *E. faecium*. After the first 2 days, a higher number of *Blastocystis* cells co-incubated with the OD^620^ = 1.0 (8.48 × 10^8^ CFU/mL) concentration of *E. faecium* was noted (p = 0.0021), and during the 5th day the OD^620^ = 1.5 (1.23 × 10^9^ CFU/mL) concentration inhibited the proliferation of the protozoan cells (p = 0.0063).

Of the four tested bacteria, *L. rhamnosus* and *L. lactis* definitely inhibited *Blastocystis* growth from the 2nd day of co-incubation, as compared to the control samples. Inhibition was perfectly visible during all 5 days when OD^620^ = 1.0 concentration (8.72 × 10^8^ CFU/mL and 8.48 × 10^8^ CFU/mL, respectively) was added and this was statistically significant (p = 0.0076 and p = 0.0383).

### Co-incubation of bacteria with *Blastocystis* ST3 axenic culture

**Fig. 2:**
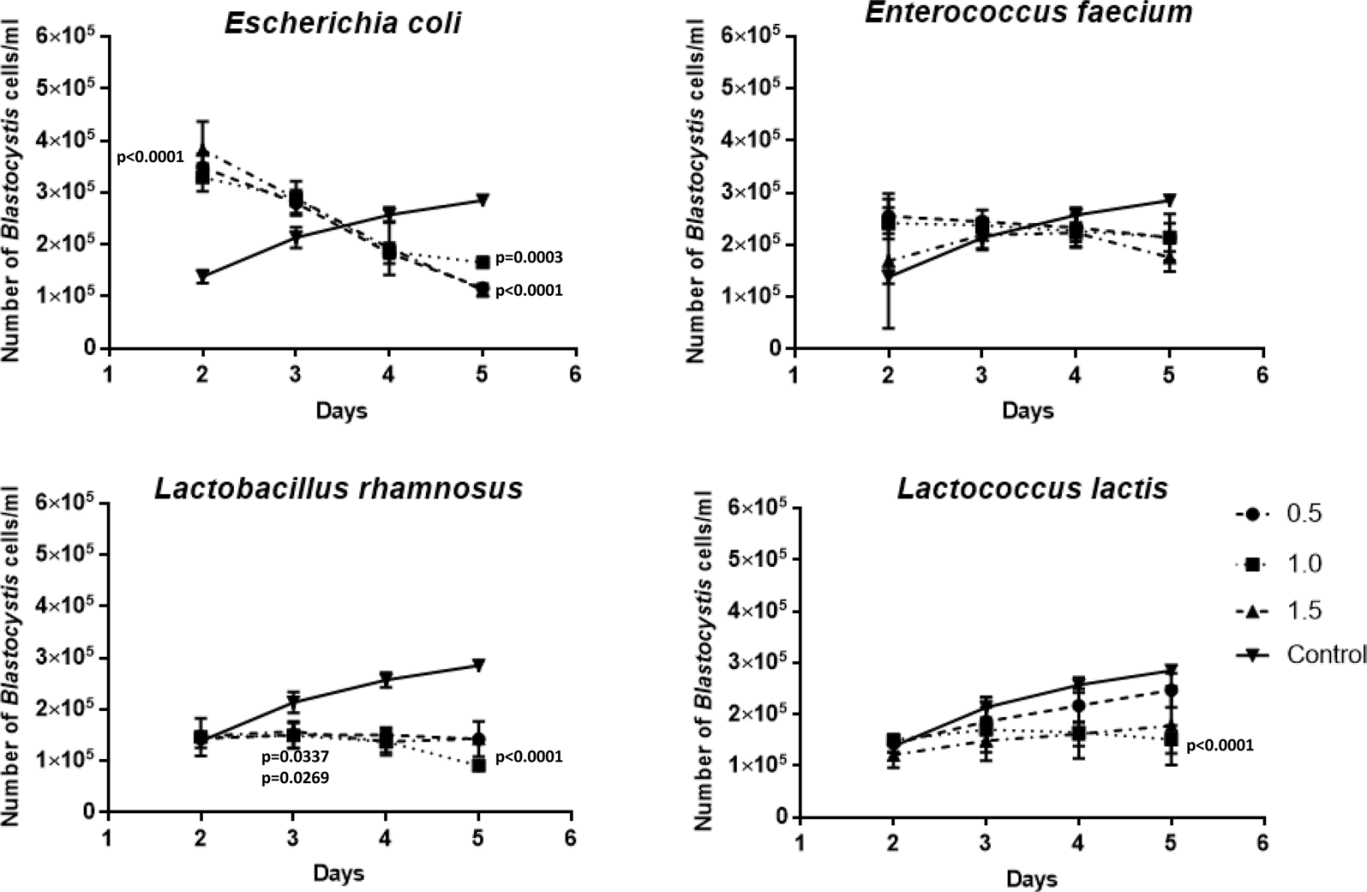
The influence of different concentrations OD^620^ = 0.5, 1.0 and 1.5 of chosen bacteria on *Blastocystis* axenic culture development according to time of co-incubation

In control samples, *Blastocystis* proliferation was observed from 2 to 5 days, but according to the xenic culture the number of protozoan cells was lower. Total viable *Blastocystis* cells in bacteria-treated cultures were counted every day starting from the 2nd day of co-incubation when the number of parasites increased significantly after the addition of different concentrations of *E. coli* (Fig. 2). *E. faecium* did not influence the *Blastocystis* significantly. A decrease in *Blastocystis* cell viability was observed after 3 days and later after co-incubation with *L. rhamnosus* and *L. lactis* (Fig. 2). Furthermore, in this case the number of *Blastocystis* cells co-incubated with *E. coli* and *E. faecium* was similar to the control samples. Also, after four and 5 days *E. coli* had a negative influence on *Blastocystis* proliferation, and that inhibition was statistically significant.

The influence of different bacterial concentrations was statistically analyzed. The number of added *E. coli* cells when optical density (OD^620^) was 0.5, 1.0 and 1.5 (4.48 × 10^8^ CFU/mL, 8.56 × 10^8^ CFU/mL, and 1.22 × 10^9^ CFU/mL, respectively) significantly influenced a higher *Blastocystis* proliferation after the first 2 days (p < 0.0001) as compared to the control sample. The number of parasites decreased on the 5th day of co-incubation with *E. coli*. In this case, all of the different concentrations (OD^620^ = 0.5, 1.0, and 1.5) again had a statistically significant influence (p < 0.0001, p = 0.0003, p < 0.0001, respectively). A similar situation with regards to *E. faecium* was observed. After the first 2 days, a higher number of *Blastocystis* cells co-incubated with the OD^620^ = 0.5 and OD^620^ = 1.0 (4.02 × 10^8^ CFU/mL, 8.48 × 10^8^ CFU/mL) concentration of *E. faecium* was noted. Moreover, during the 5th day the number of *Blastocystis* cells decreased but not significantly (Fig. 2).

In the experiment with *L. rhamnosus* and *L. lactis* the inhibition of *Blastocystis* proliferation was noted during the entire duration of the co-incubation. This was quite clearly visible during the 3rd day of co-incubation when OD^620^ = 0.5 and OD^620^ = 1.0 concentrations of *L. rhamnosus* (4.48 × 10^8^ CFU/mL and 8.72 × 10^8^ CFU/mL, respectively) were added. Statistical analysis showed significance with *p*-values lower than 0.05, p = 0.0337 and p = 0.0269, respectively. On the 5th day, all of the concentrations of *L. rhamnosus* and *L. lactis* significantly inhibited *Blastocystis* growth.

Both experiments, with xenic and axenic cultures, showed similar results with regards to *Blastocystis* inhibition by *L. rhamnosus* and *L. lactis* from the 2nd day of co-incubation with those bacteria. Also in both, co-incubation with *E. faecium* and *E. coli* showed a beneficial influence on *Blastocystis* during first 2 days. Only after 3 days did the above-mentioned bacteria start to inhibit *Blastocystis* growth in xenic and axenic culture. Usually the vacuolar form was observed. The cyst form occurred rare. In the cultures co-incubated with *E. coli* and *E. faecium* a high level of amoebic forms of *Blastocystis* was noticed.

### Co-incubation of fungi with *Blastocystis* ST3 xenic and axenic culture

**Fig. 3:**
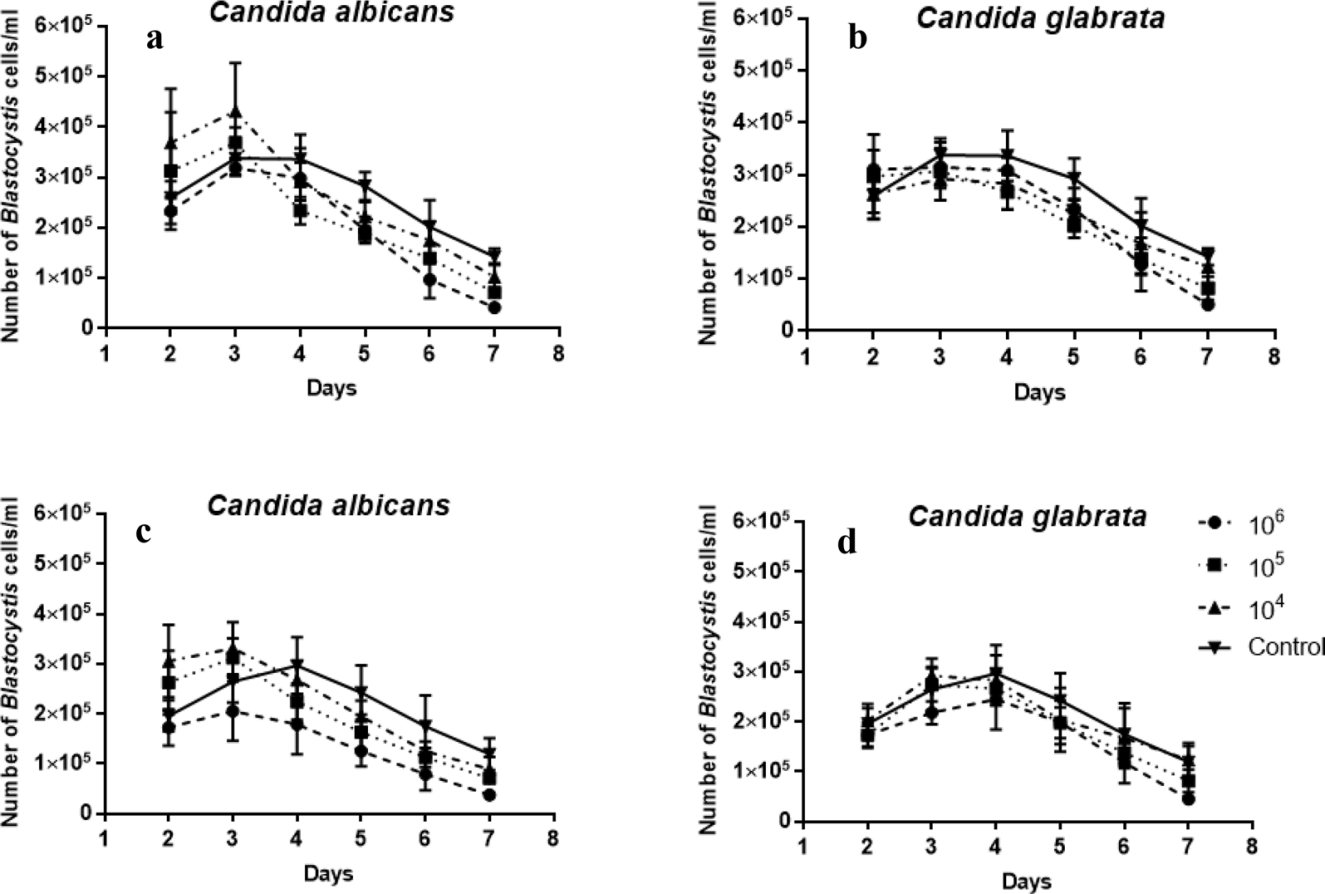
The influence of different concentrations of chosen fungi on *Blastocystis* xenic (**a**, **b**) and axenic (**c**, **d**) culture development according to time of co-incubation

Both *Candida albicans* and *Candida glabrata* showed only a modest decrease of 30% cell loss compared to the bacteria. The results of this study showed that the fungal suspension had an influence on *Blastocystis*, but the effectiveness was lower than that of bacteria (Fig. 3). The mean and standard deviation values were calculated with respect to the cell counts of the control. In control samples, *Blastocystis* proliferation was observed from 2 to 4 days. After that time it began to decrease. Total viable *Blastocystis* cells in fungi-treated cultures were counted every day starting from 2 days of co-incubation when the number of the parasite increased after the addition of 10^5^ CFU/mL and 10^6^ CFU/mL concentrations of *Candida albicans* in both xenic and axenic culture (Fig. 3a, c). During the 4th day, in both control and co-incubated samples, the number of *Blastocystis* cells started to slowly decrease. Compared to the control samples, co-incubation with both *Candida albicans* and *Candida glabrata* showed a faster decrease in *Blastocystis* proliferation (Fig. 3a, b). This was not statistically significant. A similar situation was noticed in the axenic culture experiment (Fig. 3c, d). Usually the vacuolar form occurred in the co-incubated samples. Interestingly, the amoeboid form was observed quite often as well as granular and cyst form.

### Co-incubation of cell free supernatant

**Fig. 4:**
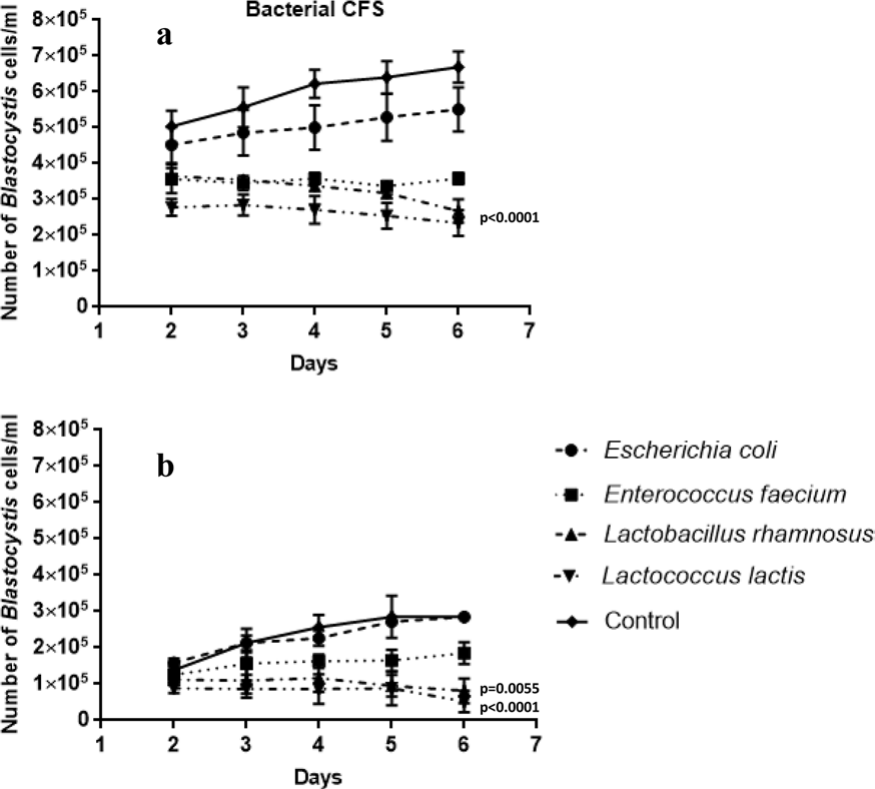
The influence of bacterial cell free supernatants (CFS) on *Blastocystis* xenic (**a**) and axenic (**b**) culture development according to time of co-incubation

**Fig. 5:**
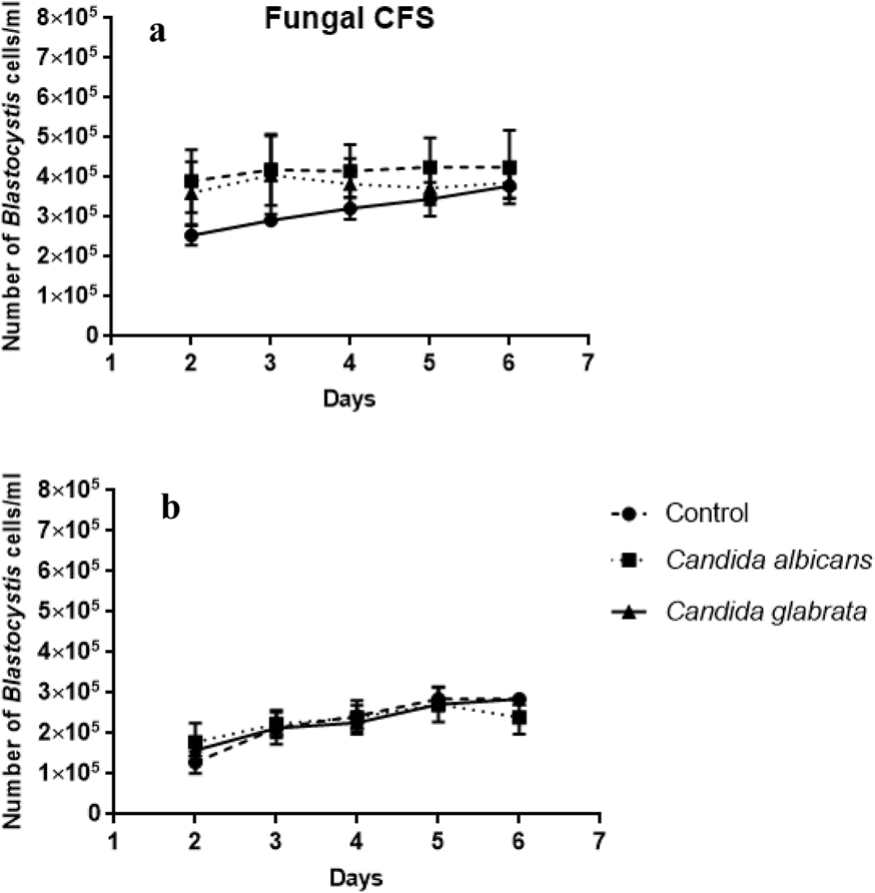
The influence of fungal cell free supernatants (CFS) on *Blastocystis* xenic (**a**) and axenic (**b**) culture development according to time of co-incubation

Only bacterial supernatants of *E. faecium*, *L. rhamnosus* and *L. lactis* inhibited *Blastocystis* proliferation in xenic culture significantly (p < 0.0001) from the 2nd day of co-incubation (Fig. 4a). The supernatant containing the metabolites of *E. coli* was effective to a lower degree. Also, in axenic culture three supernatants obtained from *E. faecium, L. rhamnosus* and *L. lactis* had a negative influence on *Blastocystis* development, but mostly it was *L. rhamnosus* and *L. lactis* (*p*-values between 0.0055 and < 0.0001) (Fig. 4b). The CFS obtained from *E. coli* had no influence on the axenic culture of *Blastocystis*. The fungal supernatants seemed not to exhibit any inhibition on *Blastocystis* growth in xenic and axenic cultures (Fig. 5a). Moreover, it showed a higher number of *Blastocystis* cells in xenic cultures (Fig. 5b) with the addition of supernatants than in control samples. Most probably the reason is a lower pH of microbiological medium in control samples.

### pH changes during co-incubation

The pH of all of the co-cultures was measured as well as cell free supernatants and microorganisms cultures on appropriate media. The pH of CFS of *E. coli* was equal to 6.5, *E. faecium*—5.45, *L. rhamnosus*—5.0, *L. lactis*—5.26, *C. albicans*—4.53 and *C. glabrata*—4.71. The pH of *E. coli* culture incubated for 2 days on TSB was equal to 6.1, *E. faecium*—5.36, *L. rhamnosus*—4.94, *L. lactis*—5.16, and of *C. albicans* culture incubated for 6 days on Sabouraud broth was equal to 4.65, *C. glabrata*—4.68.

**Fig. 6:**
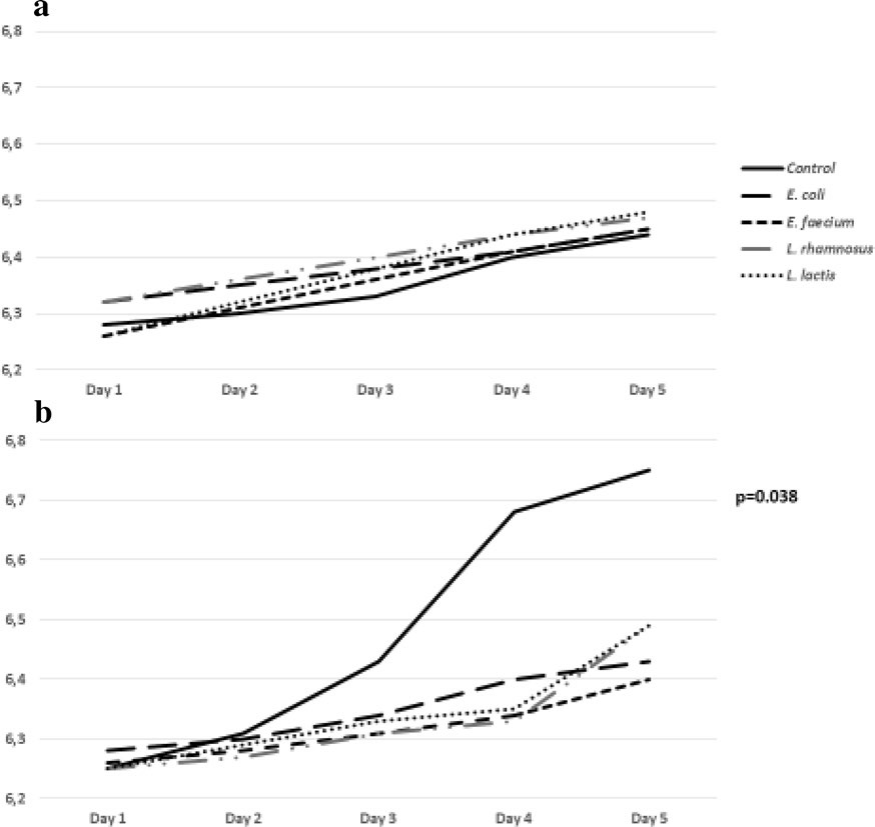
The pH changes during co-incubation of *Blastocystis* cultures with alive bacteria (**a**) and bacterial cell free supernatants (**b**)

**Fig. 7:**
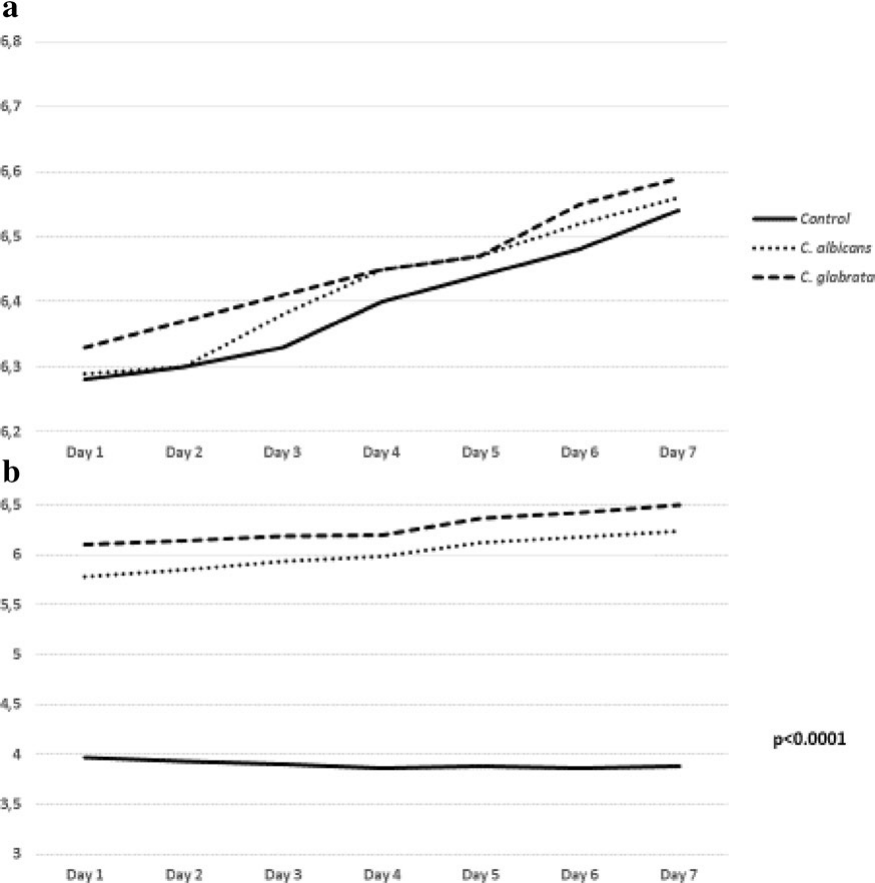
The pH changes during co-incubation of *Blastocystis* cultures with alive fungi (**a**) and fungal cell free supernatants (**b**)

The pH of *Blastocystis* cultures co-incubated with alive bacteria (Fig. 6a) ranging from 6.26 at the 1st day to 6.48 at the 5th day of co-incubation and alive fungi (Fig. 7a) ranging from 6.29 at the 1st day to 6.59 at the 7th day of co-incubation was higher regarding to the control samples (pH value from 6.28 to 6.44 at the 5th day and 6.54 at the 7th day). Also the pH of *Blastocystis* cultures co-incubated with fungal CFSs (Fig. 7b)—from 5.78 to 6.5 at the 7th day was higher regarding to the control samples (from 3.97 to 3.88 at the 7th day). Conversely, the pH of *Blastocystis* cultures co-incubated with bacterial CFSs ranging from 6.25 at the 1st day to 6.49 at the 5th day of co-incubation was lower than in control samples—from 6.25 to 6.75 (Fig. 6b).

It can be assumed that the small pH differences did not influence on *Blastocystis* proliferation significantly.

### Metronidazole control

Living lactic acid bacteria, *L. rhamnosus* and *L. lactis*, as well as their cell free supernatants had a similar effect to the MTZ control concentrations of 10 µg/mL and 5 µg/mL in xenic cultures of *Blastocystis.* Axenic culture could not be described, because of a low number of *Blastocystis* cells which nearly all died after the addition of different concentrations of MTZ. With regards to this, there were statistically significant differences between both *E. coli* and *E. faecium*, as well as *C. albicans*, *C. glabrata* and MTZ addition (p < 0.0001).

## Discussion

Natural gut microbiota plays a very important role in controlling intestinal diseases and keeping the intestines healthy. Studies have shown that intestinal microbiota could alter the *Blastocystis* [16]. Consequently, suggestions have been made that the use of the antiprotozoal drug known as metronidazole could give rise to drug resistant *Blastocystis* subtypes [31]. Moreover, metronidazole, which is the first-line treatment, has been shown to exhibit side effects and quite often a low effectiveness of this drug during eradication is noted [32]. As an alternative, we have tried to explore the potential of probiotic bacteria. We studied the response of *Blastocystis* in vitro to two different strains of the *Lactobacillaceae* family—*L. rhamnosus* and *L. lactis*, as well as *E. faecium*. In addition, we tried to define the role of *E. coli* and two fungal strains from the *Candidaceae* family in protozoan development using concentrations similar to that in a healthy human colon.

Our study shows the strong inhibitory effect of various lactic acid bacteria (LAB) at different concentrations on the proliferation of the *Blastocystis* from the beginning of co-incubation in xenic and axenic culture, whereas *E. faecium* and *E. coli* exhibited anti-proliferative activity after 4 days. In the case of co-incubation with *E. faecium* and *E. coli*, a high level of amoebic forms of *Blastocystis* was noticed. It may mean that the bacteria mentioned above are beneficial for *Blastocystis* development and confirms the previous research of Rajamanikam and Govind [33], who suggest that the amoebic form is found during optimal conditions for *Blastocystis* growth and plays a role in the exacerbation of intestinal symptoms during *Blastocystis* colonization. In our study, fungi from the *Candidaceae* family had little antiprotozoal influence, as well as forcing the vacuolar forms into cysts and granular forms. Those forms are usually observed during thermal stress, as Thergarajan et al. [34] reported in their research.

Our experiments on axenic cultures of the parasite confirmed a strong negative influence of LAB on *Blastocystis*. The cell free supernatants of bacteria were used for further investigation to determine whether the bacterial metabolites showed similar results. Sarjapuram et al. [22] reported the inhibition of other protozoan proliferation by spent media of probiotic culture. They noticed changes of the pH of microbiological media and adjusted it to eliminate its influence on *Entamoeba* growth [22]. Similarly, our study showed that not only living bacteria had a negative influence on *Blastocystis,* but also their metabolites. Zhang et al. [35] determined the optimal pH for *Blastocystis* growth to be 7.0 [35].

Our study clearly shows the inhibition of *Blastocystis* proliferation by LAB, which suggests that people using probiotic rich diets and having a stable gut microbiota are more resistant to protozoan colonization. Several previous studies revealed that the total bacterial population, as well as bacterial groups such as *Bifidobacterium* sp., *Bacteroides* spp., and *Clostridium* sp., shows a high degree of temporal stability [36–39]. However, the situation is different for the *Lactobacillus* population. The Walter et al., Vanhoutte et al., and Scanlan et al. studies of fecal samples from most human subjects showed temporal dynamics that were characterized by fluctuations and a lack of stability [36, 38, 40]. A lack of these bacteria in the large intestine caused by a poor diet, antibiotic therapy or taking drugs for gastrointestinal disorders such as proton pomp inhibitors (PPI) may influence the susceptibility to *Blastocystis* invasions [41, 42]. The interesting thing is that all of the bacterial concentrations used in this study affected the parasite proliferation. This suggests that lower bacterial CFUs may also be used for *Blastocystis* eradication. Molan in his research [18] suggested, which we validated in our work, that the factor which causes that inhibition may be the bacteria themselves or their chemical compounds. Lactobacilli increase their protective or therapeutic effect through the production of antimicrobial compounds [43], a reduction of gut pH by stimulating the lactic acid producing microflora [44], competition for binding of receptor sites that pathogens occupy and competition with pathogens for available nutrients [45, 46]. Nisin-producing *L. lactis* strains have high antimicrobial properties [47]. This study shows that lactic acid produced by the *Lactobacillaceae* family lowered the pH of the TSB medium, but not of the Jones’ medium during co-incubation with *Blastocystis*. That means the pH could not be a reason for the decline of *Blastocystis*, as has been reported about other protozoan parasites such as *Entamoeba histolytica*, *Giardia* sp. or *Eimeria* sp. [19, 21, 22, 48].

The other situation has been demonstrated by *E. faecium* and *E. coli*. Some strains of the first of these bacteria has been reported to be an effective probiotic species [22]. Our study confirmed the data from the Sarjapuram [22] research. The authors reported that *E. faecium* inhibited *Eimeria* after 24 h of co-incubation with a total bacterial CFU of 10^8^ cells/mL. *Blastocystis* ST3 seems to be more resistant to its influence. In our experiment, the more effective concentration was 1.23 × 10^9^ CFU/mL, as well as a longer incubation time—4 or 5 days. Most likely, it lasts longer because *E. faecium* produces strong antimicrobial, yet no antiprotozoal compounds, and it does not compete for enteric adherence sites. More likely, this is caused by competing for nutrients. This could be due to it being a lactic acid bacteria which colonizes differently, and competes with *Blastocystis* differently [49].

In human medicine, *E. faecium* has been used successfully in the treatment of acute diarrheal diseases and in the prevention of antibiotic—associated diarrhea [50, 51]. Starke et al., as well as Klingspor et al. in 2015, investigated the intestinal microbiota of pigs whose components are similar to human gut microorganisms [52, 53]. They showed that the probiotic bacteria *E. faecium* modifies the porcine intestinal microbiota and modulates epithelial integrity, heat shock protein as well as the proinflammatory cytokine response in intestinal cells. That could lead to the eradication of intestinal pathogens, including protozoans. Our study showed the dependence of *Blastocystis* on a fecal bacteria presence. In axenic control cultures, there were far fewer *Blastocystis* cells than in xenic culture, which means the intestinal commensal bacteria have a role in parasite development. One possible explanation for *Blastocystis* eradication by *E. faecium* may be the direct growth inhibiting effect of the probiotic on other intestinal bacteria, such as *E. coli, Clostridium* sp. or other fecal commensal microorganisms. Bednorz et al. [54] showed in their data a minor influence of *E. faecium* on the overall population of non-pathogenic *E. coli* in healthy piglets. However, this same strain has a profound effect on mucosa-adherent *E. coli*. Russo et al. and Kaper et al. reported that *E. faecium* 10415 significantly reduced pathogenic organisms, such as extraintestinal *E. coli* (ExPEC) [55, 56]. To sum up, there may be two ways of *Blastocystis* eradication by *E. faecium*: directly by cellular compounds and nutrient competition, and indirectly by killing the beneficial intestinal bacteria.

The results of our *E. coli* experiment were quite interesting. The number of *Blastocystis* cells increased, then after the 4th day began to decrease significantly. In the Ganas et al. study from 2012, *E. coli* was found to strongly support the growth of the parasite—*Histomonas meleagridis*, which may suggest it is a beneficial bacteria for protozoan parasite development [57]. That may be an explanation for the increase of *Blastocystis* proliferation, especially if *E. coli* is also an intestinal microorganism. The question remains as to why the number of *Blastocystis* cells decreased significantly after 5 days of co-incubation? One option may be the fact that the bacteria might be absorbed by *Blastocystis* at first while only low numbers of *E. coli* cells were in the incubated tubes. That supports protozoan proliferation. Another option is that *E. coli* produces endotoxins, such as lipopolysaccharides (LPS) which could negatively influence *Blastocystis* cells from inside after phagocytosis which was observed in amoebic form and destroy the parasite [57–59].

There are not many studies regarding the influence of *Candida* on protozoans. Mostly the researchers have focused on interactions between intestinal fungi and bacteria [60]. Our study focused on the interaction between *Blastocystis* and *Candida albicans* and *Candida glabrata* to determine if people who are colonized by that fungi as the natural microbiota of the human intestine are more susceptible or resistant to *Blastocystis* invasions. It can therefore be assumed that both *Candida* should to a small degree inhibit the potential pathogenic protozoan development in the intestine just as other yeasts like *Saccharomyces boulardii* [24]. Our study shows almost no inhibitory effect on *Blastocystis* by *C. glabrata* and only limited inhibition by *C. albicans* in 10^6^ cells/mL concentration. Moyes et al. [61] reported that *Candida* does not produce any toxins influencing protozoa, but produces some toxins against bacteria and epithelial cells. Also, these do not change the pH of the environment. Moreover, Konno et al. and Sherrington et al. proved *Candida* adapt to environmental pH changes [62, 63]. A minor decrease in the number of *Blastocystis* cells was most likely caused by competition between the protozoan and *Candida* for space and nutrition [64].

Our study has shown the potential of using *L. rhamnosus* and *L. lactis*, as well as *E. faecium* as probiotics against *Blastocystis* colonization. The fact that these probiotic bacterial strains are able to disrupt the cell cycle of *Blastocystis* shows a promising future in the use of probiotics for prophylactic treatment of blastocystosis, or as an additional treatment regimen in combination with standard drugs. The obtained results did not show what is the mechanism of *Blastocystis* inhibition by lactic acid bacteria. This issue requires further research.
